# CBCT-based adaptive radiotherapy and automated radiotherapy decision-making systems: development and current status

**DOI:** 10.3389/fonc.2026.1957030

**Published:** 2026-10-02

**Authors:** Shang Wang, Xin Wang

**Affiliations:** Department of Radiation Oncology, Division of Abdominal Tumor Multimodality Treatment, Cancer Center, West China Hospital, Sichuan University, Chengdu, Sichuan, China

**Keywords:** adaptive radiotherapy, artificial intelligence, automated delineation, automated radiotherapy decision-making, radiotherapy

## Abstract

Cone-beam computed tomography (CBCT)-based adaptive radiotherapy (ART) has emerged as a promising strategy for addressing inter-fractional anatomical and physiological changes during treatment. By incorporating on-treatment imaging, ART enables treatment plans to be adapted to the anatomy of the day, with the potential to improve target coverage and reduce dose exposure to organs at risk (OARs). However, routine implementation remains constrained by the limited image quality of CBCT, uncertainties in dose calculation, labour-intensive workflows and the lack of reliable criteria for determining when adaptation is clinically necessary. This review summarises the development, current status and future prospects of CBCT-based ART, with particular emphasis on artificial intelligence (AI)-assisted automated delineation and automated radiotherapy decision-making systems. CBCT is widely available on modern linear accelerators and remains the most practical imaging basis for ART in many centres. Nevertheless, poor soft-tissue contrast, noise, artefacts, field-of-view truncation and unstable Hounsfield unit values limit its performance in target delineation and dose reconstruction. Recent advances in image-quality enhancement, deformable image registration and synthetic CT generation have partially addressed these limitations. AI-based auto-segmentation models, including convolutional neural networks, Transformers, generative adversarial networks and diffusion models, have demonstrated increasingly favourable geometric accuracy and workflow efficiency. Automated decision-making systems can advise clinicians whether treatment-plan adaptation is required and how it should be implemented according to each patient’s circumstances, representing a prerequisite for more efficient ART. Current research has mainly focused on two approaches: adaptation triggered by predefined dosimetric thresholds, and artificial-intelligence-based models that learn from prior knowledge and predict potential dosimetric changes for the current patient based on previous experience. Both approaches have their respective strengths and limitations, but require further refinement, including the systematic exploration of decision thresholds. Moreover, the realisation of fully automated adaptive radiotherapy will require the integration of additional systems, including automated treatment planning and automated QA. Overall, CBCT-based ART assisted by AI is technically feasible and may improve dosimetric quality and treatment efficiency. Future progress requires improved CBCT-specific algorithms, integrated automated planning and QA, multicentre validation, standardised safety frameworks and stronger evidence of clinical benefit.

## Introduction

Modern radiotherapy has developed rapidly since its emergence in the 1990s, progressively evolving toward precision radiotherapy. Early radiotherapy primarily relied on two-dimensional simulation and simple anteroposterior or opposed-field irradiation. Target delineation was relatively crude, and dose distributions were unable to adequately balance tumor control with normal-tissue sparing. With the widespread adoption of computed tomography (CT) and advances in computerized dose-calculation techniques, three-dimensional conformal radiotherapy (3D-CRT) was gradually introduced into clinical practice during the 1990s. This approach enabled irradiation conformal to the three-dimensional shape of the tumor, substantially improving target coverage and dose distribution while reducing the dose to organs at risk. Subsequently, emerging technologies—including intensity-modulated radiotherapy (IMRT), volumetric modulated arc therapy (VMAT), image-guided radiotherapy (IGRT), stereotactic body radiotherapy (SBRT), and proton and heavy-ion radiotherapy—have further refined spatial dose distributions, increased dose conformity within target volumes, and reduced normal-tissue exposure. Consequently, radiotherapy has assumed an increasingly important role in multidisciplinary cancer treatment (1).

Since the beginning of the 21st century, the goals of radiotherapy have continued to evolve, shifting from the sole pursuit of tumor eradication toward individualized, precise, and increasingly intelligent treatment while maintaining therapeutic efficacy. Advances in imaging technologies have enabled more accurate characterization of the anatomical and functional features of tumors and surrounding normal tissues, providing a foundation for precise target delineation and individualized dose-prescription strategies. In parallel, the integration of dosimetric and biological models, radiomics, big-data analytics, and artificial intelligence (AI) has enabled clinicians to incorporate tumor stage, genetic characteristics, functional status, and treatment response when optimizing the radiation dose, fractionation regimen, and irradiation volume for individual patients (1, 2).

However, as radiotherapy planning has become increasingly precise, dynamic changes throughout the treatment course—including tumor shrinkage or progression, minor variations in patient positioning, and motion of the tumor or surrounding organs caused by respiration or digestion—have emerged as important factors affecting the accuracy of dose delivery (3, 4). If the initial treatment plan is continued without adjustment, the dose delivered to the tumor may be inadequate, or dose constraints for organs at risk (OARs) may be exceeded. These changes may compromise the therapeutic benefits of radiotherapy and increase the risk of treatment-related toxicity. To address this issue, adaptive radiotherapy (ART) was introduced as an approach for dynamically updating the treatment plan throughout the course of treatment. ART uses on-treatment imaging and, where appropriate, other clinical information to periodically or online modify the treatment plan according to the patient’s current anatomical and physiological status (5). However, conventional ART relies heavily on manual image registration, target and OAR delineation, dose reconstruction, and treatment-plan optimization. These processes are labor-intensive and time-consuming, which limits the implementation of ART in routine clinical practice. In recent years, rapid advances in AI and high-performance computing have facilitated the development of automated delineation, treatment planning, and decision-support technologies, creating new opportunities for more efficient and standardized ART workflows (6, 7). In this review, we summarize the current research status, key technologies, and future directions of cone-beam computed tomography (CBCT)-based ART and AI-assisted decision-making systems for cancer treatment.

## Scope and literature identification

This article is a state-of-the-art narrative review. Its purpose is to summarise the development, current clinical and technical status, major challenges, and future directions of CBCT-based ART, with particular emphasis on AI-assisted automated delineation and automated decision-making for treatment adaptation.

A targeted literature search was conducted in PubMed and Web of Science for studies published from database inception to July 2026. The search combined terms related to CBCT and adaptive radiotherapy, including “cone-beam computed tomography” OR “CBCT”, “adaptive radiotherapy” OR “adaptive radiation therapy” OR “online adaptive radiotherapy”, “auto-segmentation” OR “automated delineation”, “synthetic CT”, “deformable image registration”, “dose reconstruction”, “decision-making”, “adaptation trigger”, “artificial intelligence”, “machine learning”, and “deep learning”. Reference lists of relevant reviews and key original studies were also manually screened to identify additional pertinent publications.

Priority was given to English-language peer-reviewed original studies, prospective clinical studies, technical validation studies, influential reviews, and consensus or guideline documents that addressed CBCT-based ART workflows, image-quality enhancement, contour propagation or auto-segmentation, synthetic CT or dose calculation, plan adaptation, and automated decision-making. Given the rapidly evolving nature of the field, representative recent studies and landmark earlier reports were selected to provide a balanced overview.

This review does not aim to perform a quantitative synthesis or formal assessment of study quality and risk of bias. Therefore, its conclusions should be interpreted as an expert synthesis of the current evidence and technological landscape.

## Cone-beam computed tomography technology and its fundamental applications in cancer radiotherapy

Linear accelerator–integrated CBCT uses a gantry-mounted X-ray source and a two-dimensional detector to acquire projection images as the gantry rotates around the patient. These projections are reconstructed into three-dimensional volumetric images (8). Unlike conventional planning CT, CBCT enables on-treatment imaging with the patient positioned on the treatment couch, providing anatomical information for image guidance and assessment of interfraction changes (9). CBCT is widely used in image-guided radiotherapy (IGRT), particularly in centers equipped with contemporary linear accelerators. However, its availability, acquisition protocols, and clinical applications vary across treatment platforms, anatomical sites, and institutional resources. Depending on image quality and soft-tissue contrast, pretreatment CBCT can visualize relevant anatomical structures and support assessment of their positions and spatial relationships relative to the planning CT.

In routine clinical practice, pretreatment CBCT images are registered to the planning CT using bony landmarks, soft-tissue targets, or other relevant structures. The resulting positional discrepancies guide treatment couch corrections. Translational corrections are commonly applied, whereas rotational corrections depend on couch capabilities and clinical protocols (10). This approach primarily compensates for rigid setup errors and can improve geometric alignment between the planned and treatment positions. It does not, however, directly correct anatomical deformation or routinely involve beam reoptimization.

Conventional CBCT-guided setup correction generally retains the original treatment plan and optimization objectives. Although it can compensate for global positional shifts, it may not adequately address tumor regression or progression, organ filling changes, or nonrigid deformation. Under these conditions, the dose distribution calculated on the planning CT may no longer accurately represent the dose delivered to the current anatomy. Target coverage may deteriorate, and doses to OARs may increase or exceed planning constraints. The magnitude and clinical relevance of these deviations depend on the anatomical site, the extent of anatomical change, and the robustness of the original plan.

These limitations provide a rationale for CBCT-based ART, in which on-treatment anatomical information is used to assess whether plan modification is warranted. Rather than assuming that every anatomical change requires adaptation, this approach seeks to identify clinically relevant deviations and support appropriate plan adjustment. Its implementation depends on adequate image quality, reliable contouring and dose calculation, and appropriate quality assurance. Although CBCT-based adaptation has demonstrated technical and dosimetric feasibility in selected settings, improvements in patient-centered clinical outcomes require further prospective validation.

## CBCT-based adaptive radiotherapy for cancer

The core principle of ART is to modify the treatment plan in response to changes occurring during treatment, with the aim of maintaining target coverage and limiting normal-tissue exposure in accordance with predefined treatment objectives. Adaptation primarily addresses anatomical changes, although physiological and biological information may also inform treatment modification in selected or investigational settings. Based on its timing, ART is commonly classified as offline or online (11). Offline ART uses imaging and other information acquired during previous fractions to guide replanning, with the revised plan implemented in subsequent fractions. Its timing may follow a predefined schedule or be prompted by clinically relevant changes. Online ART involves updating or selecting a treatment plan using on-treatment imaging while the patient remains on the treatment couch, followed by delivery during the same session. ART can also be categorized according to the information guiding adaptation. Anatomical adaptation addresses changes in tumor and OAR position, volume, and shape, whereas biological adaptation uses functional or metabolic imaging, or other biological indicators, to inform changes in dose prescription or distribution. Biological adaptation remains less established in routine practice and is not generally supported by CBCT alone. Advances in CBCT imaging and integrated treatment platforms have enabled the clinical implementation of online anatomical adaptation in selected settings.

A representative CBCT-based online ART workflow begins with pretreatment CBCT acquisition and registration to the planning CT to assess anatomical alignment and guide setup correction where appropriate. Rigid registration estimates translational and rotational offsets but cannot account for nonrigid deformation. Deformable image registration (DIR), automated segmentation, or a combination of these methods may then be used to generate target and OAR contours, followed by clinical review and correction as required. The original plan is recalculated on an image dataset suitable for dose calculation and representative of the current anatomy, such as a corrected CBCT or synthetic CT (sCT). Target coverage and OAR doses are evaluated against predefined criteria. Depending on the platform and clinical protocol, an adapted plan may be generated routinely for comparison or only when specified criteria are met. The selected plan undergoes clinical review and appropriate quality assurance (QA), which may include independent dose verification and automated integrity checks. Dose–volume histogram (DVH) metrics support plan evaluation but do not replace these QA procedures. Additional verification imaging may be required to assess anatomical changes occurring during adaptation before treatment delivery. The feasibility and duration of this workflow depend on the anatomical site, platform capabilities, contour-editing requirements, and local review procedures. **Table 1** summarizes selected studies of CBCT-based ART (12–15).Studies involving prostate, head and neck, lung, abdominal, and pelvic cancers have reported improvements in selected dosimetric endpoints, although the magnitude and consistency of these benefits vary across sites and study designs (16–21). These findings primarily support technical and dosimetric feasibility rather than establish improvements in patient-centered clinical outcomes.

**Table 1:** Selected studies of CBCT-based ART.

Study	Site/No. of patients	Imaging platform	Adaptive strategy	Primary outcomes	Workflow time
Branco et al., 2023 (24)	Cervical cancer/n=15	Varian Ethos^®^ system	Manual	bladder and rectum D50% decreased by 37.7% and 35.8%	less than 20 min (range: 1–29 min)
Fischer et al., 2025 (13)	Bladder cancer/n=160 fractions	Varian Ethos^®^ system	automated	enhanced target dose coverage(median D98% 98.1%), reduced OAR doses	Median treatment time was 14 min
Wurschi et al., 2025 (14)	prostate cancer/n=7	Varian Ethos^®^ system	automated	target coverage for CTV1 (V100%, p = 0.01), PTV1 (D98%, p < 0.001), PTV2 boost (D98%, p < 0.001) in Wilcoxon signed-rank tests. OAR dose reduction was limited, with a small improvement in bladder V40Gy (p = 0.02)	Mean treatment time was 30:17 (± 05:49 SD) minutes per fraction
Olberg et al., 2025 (15)	pancreatic cancer/n=10	Varian Ethos^®^ system	automated	PTV prescription coverage increased by 23.3 ± 9.4% through plan adaptation. Point doses to the stomach were reduced by 10.2 ± 9.3%	/
Åström et al., 2022 (12)	anal cancer/n=23	Varian Ethos^®^ system	automated	V45Gy of 11.4% and bladder V35Gy of 16.1%	median oART procedure time was 23 min

Despite these advances, several challenges continue to limit broader implementation. One concerns CBCT image quality and its suitability for dose calculation. Compared with conventional planning CT, CBCT is generally more susceptible to scatter, noise, and reconstruction artifacts because of its acquisition geometry and system characteristics. Soft-tissue contrast may be limited, and motion artifacts or field-of-view truncation can further impair image quality. The severity of these limitations varies across platforms, acquisition protocols, patient sizes, and anatomical sites. CBCT numbers, expressed in Hounsfield units (HU), may also differ from those obtained with planning CT and may vary with imaging conditions (22). These uncertainties can affect contour delineation and the conversion of image intensities into the density information required for dose calculation. Consequently, uncorrected CBCT images should not be assumed to provide sufficiently accurate dose estimates without platform- and site-specific validation. Appropriate correction, calibration, or alternative image-generation methods are therefore important components of many CBCT-based ART workflows.

A second challenge is the time and staffing required for online adaptation. Image registration, contour review, plan recalculation or reoptimization, dose evaluation, and QA must be coordinated while the patient remains on the treatment couch. Workflows requiring extensive manual intervention can substantially prolong treatment sessions, although the duration varies with case complexity, platform integration, and staff experience. Longer sessions may increase patient discomfort and the likelihood of intrafraction anatomical changes, while also reducing treatment-unit capacity. These demands can constrain implementation, particularly in centers with limited staffing or infrastructure (23, 24). Automation may reduce some of these demands, but its practical benefit depends on the time required for review, correction, and management of workflow exceptions.

Several strategies have been investigated to improve CBCT image quality and dose-calculation accuracy. Scatter correction and iterative reconstruction can reduce artifacts and improve image quality, although their effectiveness depends on the acquisition system and imaging conditions. Methods for improving the suitability of CBCT for dose calculation include protocol-specific HU-to-density calibration, tissue-specific density overrides, and deformation of the planning CT to match the anatomy depicted on CBCT (25). These approaches address different sources of uncertainty and are not interchangeable. Their suitability requires validation for the intended anatomical site, treatment technique, and clinical workflow.

Deep learning provides another approach to generating sCT images from CBCT. These methods aim to preserve the anatomy depicted on CBCT while producing CT-like image intensities suitable for dose calculation. Depending on the model, planning CT information may be used during training, provided as an additional input, or not required during inference. Studies have reported reduced dose-calculation discrepancies relative to uncorrected CBCT in selected datasets and anatomical sites (26, 27). However, accurate image appearance does not necessarily imply accurate tissue-density representation. Performance may deteriorate in the presence of substantial anatomical changes, uncommon imaging artifacts, or data that differ from the training distribution. Clinical use therefore requires dosimetric validation and checks for anatomical fidelity, rather than reliance on image-similarity metrics alone.

AI is also being investigated and implemented to support workflow automation. Deep-learning segmentation models can generate OAR contours and, in selected applications, target contours or refinements of propagated contours. Their accuracy and potential time savings depend on the anatomical site, image quality, structure type, and extent of manual correction required. Target delineation may additionally require clinical information that is not available from CBCT alone. Automated optimization and knowledge-based planning (KBP) can support plan generation using predefined objectives and constraints, but the resulting plans still require assessment of clinical acceptability (28). For clarity, this review distinguishes four related terms. Decision support refers to systems that provide information or recommendations to assist clinicians, who retain responsibility for the final decision. Adaptation triggering refers to the identification of predefined anatomical, dosimetric, or other criteria that prompt assessment for possible adaptation; a trigger does not necessarily mandate replanning. Automated decision-making is used as an umbrella term for computational methods that generate an adaptation recommendation or plan-selection output, with the extent of human oversight specified for each application. Fully automated decision-making refers to the selection and authorization of an adaptation action without case-specific human review before implementation. Accordingly, a workflow that automatically generates contours and treatment plans but requires clinician approval is not considered fully automated decision-making. Rule-based and machine-learning approaches have been explored to identify adaptation triggers or recommend plan selection using daily anatomical and dosimetric information (29). Systems that retain clinician approval are described here as decision-support systems, even when their underlying analyses are automated. Their value should be assessed in terms of recommendation accuracy, workflow efficiency, safety, and clinical relevance.

Although CBCT-based online ART is clinically available on selected platforms, its adoption and level of automation remain heterogeneous. Differences in imaging protocols, software, staffing, and patient populations may affect workflow performance and the generalizability of AI models. Errors in sCT generation, DIR, and contour propagation may influence downstream dose evaluation and adaptation recommendations, particularly when anatomical changes are substantial. Uncertainties in DIR also require specific consideration when it is used for deformable dose accumulation. Validation methods and acceptance criteria are not yet consistently harmonized across platforms and applications. Increasing automation therefore requires robust QA, traceable outputs, clear clinical responsibilities, and fallback procedures for unreliable or incomplete results. The strongest current evidence supports technical feasibility and improvements in selected dosimetric endpoints. Prospective evidence demonstrating consistent improvements in toxicity, quality of life, local control, or survival remains limited, particularly for automated adaptation decisions. Further progress will require platform- and site-specific validation, standardized reporting, and multicenter prospective studies that assess both workflow performance and patient-centered outcomes (29, 30).

## CBCT-based automated workflows and automated decision-making systems

Advances in AI have supported substantial progress in automated segmentation of target volumes and OARs on planning CT and magnetic resonance imaging (MRI). Studies commonly evaluate geometric agreement using the Dice similarity coefficient (DSC), Hausdorff distance (HD), and surface-distance metrics. For example, Peng et al. reported DSC, 95th-percentile HD (HD95), and average surface distance (ASD) values for automated cervical cancer contouring. For postoperative radiotherapy (pRT), the respective values were 0.80, 5.85 mm, and 0.95 mm for clinical target volume 1 (CTV1), and 0.84, 4.88 mm, and 0.73 mm for CTV2. For definitive radiotherapy (dRT), the corresponding values were 0.84, 6.58 mm, and 0.89 mm for the primary gross tumor volume (GTVp); 0.85, 5.36 mm, and 1.35 mm for CTV1; and 0.84, 4.09 mm, and 0.73 mm for CTV2 (31, 32). These results describe geometric agreement within the study setting but do not, by themselves, establish dosimetric acceptability or suitability for use without clinician review.

Automated segmentation tools have been incorporated into some commercial treatment-planning systems and institutional workflows, particularly for OAR delineation during initial planning. Applications have been reported in head and neck, pelvic, and breast radiotherapy, although performance and clinical adoption vary by structure, platform, and institution (33, 34). These tools can reduce manual contouring time in selected settings, but the net benefit depends on the extent of review and correction required. Target delineation presents additional challenges because clinical target definitions may depend on disease extent, patterns of microscopic spread, and information not directly visible on the input images.

For CBCT-based online ART, segmentation must represent the anatomy at the time of treatment rather than rely solely on the initial planning images. This may be achieved through direct CBCT segmentation, propagation of planning contours using DIR, or hybrid methods, with clinical review as appropriate. Compared with planning CT or MRI, CBCT may provide less distinct soft-tissue boundaries and may be affected by noise, artifacts, field-of-view truncation, and variable image intensities. The severity of these limitations depends on the treatment platform, acquisition protocol, and anatomical site (8, 25). These factors complicate model development and may reduce segmentation reliability, particularly for poorly visualized structures.

Although CBCT-based automated segmentation is used in selected online ART platforms, its performance is not uniform across structures and clinical settings. External validation across institutions, imaging protocols, and patient populations remains important. Respiratory motion, changes in organ filling, and tumor regression can produce substantial anatomical variation, increasing the risk of contour omission or inclusion of adjacent tissues. Geometric metrics alone may not adequately capture the clinical consequences of these errors, especially near steep dose gradients. Evaluation should therefore consider dosimetric effects, clinician-rated acceptability, correction time, and failure rates in addition to geometric agreement. Improving segmentation robustness and identifying unreliable outputs remain important priorities for CBCT-based ART (22, 26, 27).

Reliable segmentation does not, however, establish that every patient requires replanning before every fraction. Daily online adaptation is feasible in selected clinical workflows, but its incremental value and resource requirements vary by disease site, fractionation, anatomical variability, and institutional capacity. In some patients, the original plan may remain acceptable despite modest anatomical changes. In others, small changes near critical structures may have clinically relevant dosimetric consequences. Apparent anatomical stability alone is therefore insufficient to determine whether adaptation is unnecessary. Decision support should help clinicians identify whether adaptation is warranted, when it should occur, and which aspects of the plan require modification. Such an approach may help allocate resources according to clinical need, although its efficiency and safety require validation (6, 35, 36).

A dosimetry-informed decision-support system can combine target coverage, OAR exposure, anatomical changes, and relevant clinical risk factors. Using the current CBCT or a validated dose-calculation dataset derived from it, together with reviewed contours, the system can estimate the dose distribution that would result from delivering the original plan to the current anatomy. This is a pretreatment dose estimate, not a direct measurement of the dose actually delivered. Its accuracy depends on image quality, density representation, contour reliability, and the extent of anatomical change between imaging and treatment.

Where validated methods are available, estimated doses from previous fractions may be mapped to a common reference geometry using DIR to support cumulative dose assessment. However, deformable dose accumulation introduces additional uncertainty, particularly in regions affected by substantial deformation, tissue appearance or disappearance, and ambiguous anatomical correspondence. If the current fraction has not yet been delivered, adding its predicted dose to estimates from previous fractions yields a projected cumulative dose through the current fraction, rather than the dose already delivered. Predictions of end-of-course dose require further assumptions about the remaining fractions. These dose time frames and their associated uncertainties should be explicitly distinguished. Cumulative dose assessment may inform adaptation, but it is not a prerequisite for every online ART workflow.

The system can extract relevant DVH metrics, such as target D95 and D98, representing the doses received by 95% and 98% of the target volume, respectively. OAR metrics may include Vx, the volume or percentage receiving at least a specified dose x; maximum dose (Dmax); and mean dose (Dmean). The units, volume definitions, and dose time frame should be specified. Where appropriate, protocol-defined near-maximum dose metrics may be preferable to a single-voxel Dmax. These measures should be compared with disease-site- and fractionation-specific requirements to identify potential target undercoverage or excessive OAR exposure (37, 38). A deviation should prompt an assessment of its clinical relevance and reliability rather than automatically mandate replanning.Sun et al. investigated an RTP-Net–based AI model to support adaptation decisions in cervical cancer radiotherapy. The weighted model achieved a reported prediction accuracy of 0.855, compared with 0.795 for physician consensus. Precision was 0.917 versus 0.925, and recall was 0.887 versus 0.790 (6).

Current decision-support approaches can be broadly divided into rule-based and data-driven methods. Rule-based systems apply explicit thresholds derived from clinical protocols, prescription requirements, and OAR constraints. Adaptation triggers should reflect clinically meaningful deviations rather than arbitrary percentage changes. Each trigger should specify whether it concerns the current fraction, estimated accumulated dose, or projected end-of-course dose, together with any assumptions about subsequent treatment. Candidate thresholds should be evaluated against clinician-adjudicated dosimetric assessments in representative CBCT datasets. Sensitivity analyses should assess the effects of plausible errors in contouring, dose calculation, and registration. Repeated observations may help distinguish transient variation from persistent deterioration, but should not delay review of an immediately concerning finding.

Data-driven systems use machine learning or deep learning to predict dosimetric deviations, classify a predefined need for adaptation, or support plan selection. These tasks should be distinguished from predicting the clinical benefit of replanning. A model trained on clinician decisions learns to reproduce those decisions, whereas a model trained on dosimetric criteria predicts the corresponding dosimetric endpoint. Neither necessarily identifies the treatment strategy that will improve toxicity, tumor control, or survival. Claims about the outcomes of alternative adaptation strategies require evidence beyond prediction accuracy in retrospective datasets. Decision thresholds for predictive systems should be selected using development data and clinically justified trade-offs between false-negative and false-positive recommendations, then fixed before independent testing. Data partitioning should occur at the patient level to prevent information leakage between fractions from the same patient. Evaluation should include threshold-specific sensitivity and specificity, predictive values, and calibration when probabilistic outputs are provided. External validation across CBCT platforms and institutions is important for assessing generalizability. Prospective silent-mode evaluation, in which recommendations are recorded without influencing treatment, can help characterize performance before interventional clinical evaluation. Dataset representativeness, reference-label quality, and model interpretability remain important limitations; models intended to predict clinical outcomes additionally require adequate follow-up data (38, 39).

Clinical implementation requires integration of decision support with CBCT acquisition, segmentation, dose calculation, planning, and QA, while preserving clear clinical responsibilities (39). A realistic and feasible objective is the development of a ‘semi-automated closed-loop’ workflow. Based on the daily CBCT and previously accumulated dose, the system would automatically complete data processing and risk assessment, provide explicit recommendations—such as ‘adaptation recommended’ or ‘continuation of the original treatment plan recommended’—and report changes in key dosimetric metrics together with potential risk alerts. Clinicians could then review the system output and make the final decision within a few minutes. This approach could substantially reduce decision-making time while preserving the role of clinical experience and individualised judgement, thereby promoting clinician confidence and acceptance during the early stages of implementation. With the accumulation of data and iterative refinement of automated decision-making systems, the ultimate objective may be to achieve genuinely fully automated decision-making, either without direct clinician involvement or with clinician supervision but without the need for final decision confirmation (The workflow for automated adaptive radiotherapy is shown in **Figure 1**) (40). A semi-automated workflow should report the triggering metric, its dose time frame and relevant quality flags alongside each recommendation. False-positive recommendations may cause unnecessary replanning, QA workload and longer on-couch time, whereas false negatives may miss clinically relevant target undercoverage or OAR overdosing. Threshold selection should therefore reflect endpoint-specific clinical consequences rather than overall accuracy alone. Borderline results or failed image, contour or registration checks should prompt clinician review instead of automatic adaptation or continuation. Apparent dose deterioration should also be assessed for correctable setup or preparation errors before replanning.

**Figure 1:**
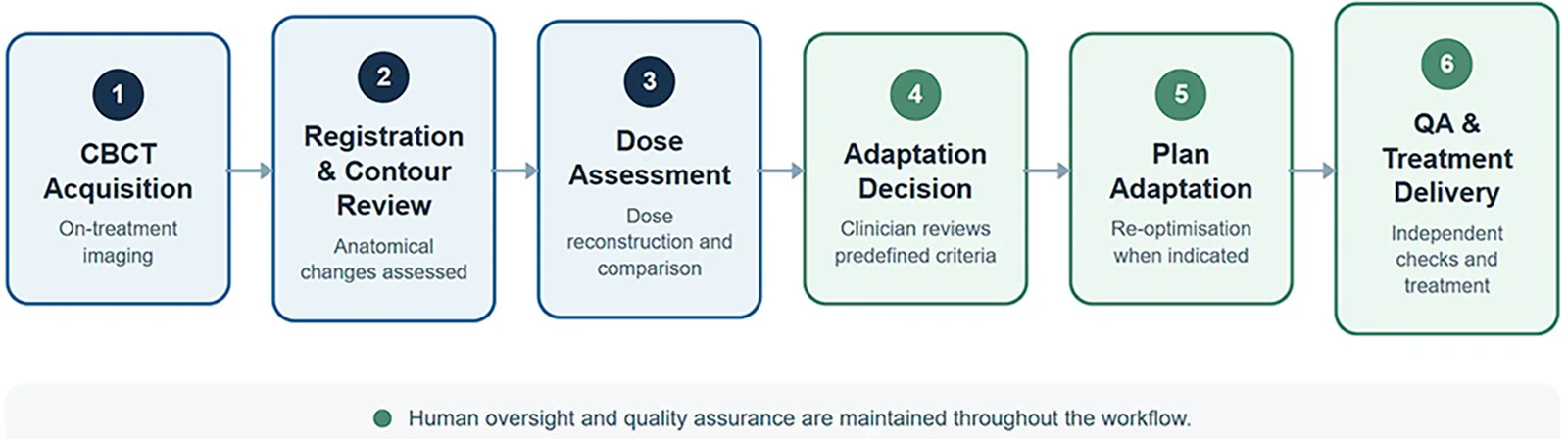
The workflow for automated adaptive radiotherapy.

Looking ahead, continued maturation of CBCT image-quality enhancement techniques, synthetic CT generation methods and CBCT-based automated delineation models, combined with automated decision-making systems that integrate rule-based and data-driven approaches, may enable adaptive radiotherapy to transition from exploratory use to routine clinical practice. At the same time, widespread implementation of ART and automated decision-making systems is expected to drive the evolution of radiotherapy quality-control frameworks, facilitate more rigorous quantification of the relationship between dosimetry and clinical outcomes, and provide a foundation for genuinely intelligent radiotherapy (40).

## Discussion

CBCT-based automated delineation and decision-support technologies have demonstrated technical feasibility, although their clinical maturity varies by application, anatomical site, and treatment platform. Studies have reported improvements in geometric agreement and selected dosimetric endpoints, with potential reductions in manual workload and treatment-planning time (31). Some centers have incorporated CT- and MRI-based automated delineation into routine initial treatment planning as supportive tools (20, 34). CBCT-based online adaptive workflows have also entered clinical practice on selected commercial platforms. However, their adoption remains uneven, and AI-assisted adaptation decisions generally require clinician review. Broader implementation requires further validation of workflow reliability, resource requirements, and clinical utility (41).

As discussed above, limitations in CBCT image quality remain an important barrier to its wider application in ART. Nevertheless, integrated CBCT is available on many contemporary linear accelerators, whereas access to CT-on-rails and MRI-linac systems is more limited and varies across institutions and regions. Although conventional CBCT generally has lower soft-tissue contrast than planning CT or MRI and less stable HU values than planning CT, its integration into treatment units makes it a practical imaging foundation for ART in many settings. This accessibility continues to motivate the development of CBCT-based adaptive workflows and automated delineation methods despite the associated imaging challenges.

In the longer term, wider access to integrated CT-guided and MRI-guided treatment platforms may provide improved imaging for selected ART applications. However, the core workflow components—including contouring, dose assessment, plan adaptation, and decision support—are shared across imaging modalities. Methods developed for CBCT may therefore inform applications on other platforms, although transfer requires modality-specific adaptation and validation. The role of sCT generation also differs: CT-guided systems may provide images directly suitable for dose calculation, whereas MRI-guided workflows require methods to derive the necessary density information. Research across these platforms may support methodological refinement, giving CBCT-based studies both immediate practical relevance and broader value for image-guided ART.

AI is an important enabling technology for ART and decision support. Approaches have expanded from conventional machine-learning models using handcrafted features to convolutional neural networks, including U-Net, and more recent architectures such as transformers, generative adversarial networks (GANs), diffusion models, and multimodal foundation models. These developments have expanded modeling capabilities, but improvements in generalizability and training stability are not guaranteed and require application-specific evaluation. In radiotherapy, selected studies have reported improved segmentation, reduced sCT dose-calculation errors, and more effective modeling of dose–anatomy relationships. Automated contours may approach expert reference contours for some structures on planning CT or MRI, but performance remains variable, particularly on CBCT. Future research should emphasize CBCT-specific model design, data augmentation, and cross-device domain adaptation to improve robustness in clinical settings (42–45).

This review focuses on two AI-assisted components: automated delineation and decision support. These components alone are insufficient to support a complete adaptive workflow. An integrated ART system also requires treatment-plan generation and optimization using updated contours and dose constraints; plan-quality evaluation, including DVH assessment and hotspot review; dose verification using measurements or independent calculations, as appropriate; and QA addressing equipment performance and workflow integrity. Progress has been made through KBP, dose-prediction networks, and automated QA alerts. Although some commercial platforms integrate several of these functions, interoperability and transferability across institutions and equipment remain limited (45–47).

Increasing automation places greater demands on quality and safety management. Automated systems may improve efficiency, but errors can propagate across interconnected workflow components. QA should therefore be developed alongside automation and include screening for poor image quality and registration failures, alerts for unexpected dosimetric results, and assessment of output uncertainty where feasible (48–50). Validation must extend beyond individual algorithms to the complete workflow, covering CBCT acquisition, registration, contour propagation, sCT generation, dose calculation, plan optimization, and adaptation decisions. End-to-end testing should use representative clinical cases, including challenging scenarios. Contour review, independent dose verification, adapted-plan checks, and failure detection should be incorporated as appropriate before treatment delivery. Human oversight remains particularly important when image quality is poor, anatomical changes are substantial, or adaptation recommendations are borderline. Audit trails should record model outputs, manual edits, QA results, and final clinical decisions. Prospective safety assessment and ongoing monitoring are also needed to identify failures during clinical use.

Clinical translation must account for differences in equipment, staffing, and case mix across centers. Much of the published evidence comes from institutions with advanced infrastructure and established multidisciplinary teams. Results obtained in these settings may not directly generalize to centers with fewer resources or different clinical workflows. Broader implementation therefore requires evaluation of staffing needs, training, treatment capacity, and local technical capabilities.

Geometric accuracy and clinical utility should be treated as distinct endpoints. High DSC values or favorable boundary metrics do not necessarily indicate clinically acceptable contours or reliable adaptation decisions, particularly for small structures or organs near steep dose gradients. Similarly, dosimetric improvements do not automatically translate into reduced toxicity, improved tumor control, or longer survival. Claims of clinical benefit therefore require prospective validation using patient-centered outcomes.

Overall, current evidence supports the technical feasibility of AI-assisted CBCT-based ART, with improvements in selected dosimetric endpoints and potential workflow efficiencies in specific settings. However, robust prospective evidence demonstrating reduced toxicity, improved quality of life, or better disease control remains limited, particularly for AI-assisted adaptation decisions. Prospective multicenter studies with clinically meaningful endpoints and rigorous safety assessment are needed to establish its clinical value. Continued advances in imaging, algorithms, and workflow integration may support broader adoption. Across CBCT-, CT-, and MRI-guided platforms, integrating automated delineation, planning, decision support, dose verification, and QA may enable more efficient and reproducible adaptive treatment. The appropriate level of automation should be determined by demonstrated safety and clinical utility, with human oversight retained where needed.
